# Awareness and Readiness To Implement the Pradhan Mantri Jan Arogya Yojana: A Cross-Sectional Study Among Healthcare Workers of a Tertiary Care Hospital in Eastern India

**DOI:** 10.7759/cureus.24574

**Published:** 2022-04-28

**Authors:** Santosh K Nirala, Purushottam Kumar, Bijaya N Naik, Sanjay Pandey, Chandramani Singh, Rajath Rao, Mohit Bhardwaj

**Affiliations:** 1 Community and Family Medicine, All India Institute of Medical Sciences, Patna, Patna, IND; 2 Community Medicine, Government Medical College & Hospital, Aurangabad, Aurangabad, IND

**Keywords:** india, health insurance, healthcare workers, readiness, awareness, ayushman bharat, pmjay

## Abstract

Background

The Indian government announced "Ayushman Bharat" for a New India 2022 during the 2018-19 parliament budget sessions, which includes the national health protection scheme presently known as "Pradhan Mantri Jan Arogya Yojana (PMJAY)" to facilitate access to secondary and tertiary healthcare services. This study aimed to see how well healthcare workers (HCWs) understood the PMJAY and how prepared they were to administer it.

Materials and methods

With an anticipated sample size of 411, this hospital-based analytical, cross-sectional study was done among treating faculty, resident doctors, and nursing officers as study participants. Participants completed a self-administered, pre-tested, semi-structured questionnaire to determine their level of awareness and readiness to adopt PMJAY. SPSS Version 22 software (SPSS Inc., Chicago, IL, USA) was used to analyze the data.

Results

The overall mean (SD) awareness score and mean readiness score among HCWs were found to be 5.52 (1.82) and 18.49 (4.5), respectively. There was a significantly high awareness score among doctors compared to nursing officers. The relation between awareness score and readiness score showed a weak positive significant correlation (r=0.174, p=0.001). The linear regression model demonstrated an increase of 0.432 units in readiness for every unit increase in awareness score.

Conclusion

The doctor's mean awareness score was little over half of the maximum attainable score. Faculty members were more aware of the scheme than the residents and nursing officers. The readiness to implement PMJAY improves as the awareness grows. Frequent workshops on PMJAY for stakeholders are required for better readiness.

## Introduction

The WHO 2019 theme was "Universal Health Coverage (UHC) - Everyone and Everywhere," which envisions universal access to healthcare services free of charge. UHC is also a significant component of the Sustainable Development Goals of the United Nations [1]. Due to financial constraints, it was estimated that around 6% of India's population would be unable to obtain medical treatment. Even if they opt to seek medical attention, the costs are high, further impoverishing them. The vast majority of Indians, over 85%, are uninsured [2].

In the year 2008, the Government of India came up with Rashtriya Swasthya Bima Yojana to cadre the unorganized sector workers who fell either below or slightly above the poverty line. A maximum amount of INR 30,000 was provided for the entire family. Later in 2013, the Government under the Ministry of Finance came up with the Aam Admi Bima Yojana, which targeted only low-income families of India who were engaged in 48 vocations with coverage of INR 30,000 per year. Similarly, the Government of India runs the Employees' State Insurance scheme and Central Government Health scheme at present [3,4].

The Indian Government launched "Ayushman Bharat for a New India 2022" during the 2018-19 parliamentary budget hearings. Two significant projects are the construction of "Health and Wellness Centres (HWC)" to improve primary care and the "National Health Protection Scheme," now known as "Pradhan Mantri Jan Arogya Yojana (PMJAY)," to provide access to intermediate and tertiary healthcare. This effort intends to give about 500 million financially disadvantaged Indians financial stability and prevent 50-60 million Indians from becoming impoverished due to escalating healthcare costs. Bihar is one of the states where the PMJAY is being implemented (PMJAY) [5-7].

It is critical to keep an eye on the program frequently to ensure that it is being implemented properly. The institution must have adequate resources for the scheme's implementation, delivery, and monitoring for it to succeed. A thorough analysis of appropriate infrastructure in institutions and an assessment of readiness among healthcare workers (HCWs) are required to ensure the program's success. HCWs must be informed of administrative and programmatic components and the impact of PMJAY on the Indian health system to ensure optimal preparation [5-9].

In this context, we planned a study to measure HCWs' knowledge and readiness about PMJAY in a tertiary care health facility in East India. This aids in determining the level of awareness and readiness among HCWs and the institution's preparation of the next measures for a smooth implementation of PMJAY.

Study objectives

To assess the level of awareness and readiness in implementing PMJAY among the HCWs in East India's tertiary care health facility. The study also aims to correlate the awareness and readiness scores among the HCWs.

## Materials and methods

Study design, duration, and setting

This hospital-based, cross-sectional study was conducted for a duration of four months (October 2021 to February 2022) at All India Institute of Medical Sciences, Patna, an institute of national importance under the Ministry of Health and Family Welfare, Government of India, with a 750-bed capacity catering the population of Bihar and neighboring states of East India by providing tertiary care. The sampling frame of our study was faculty, residents, and nursing staff of AIIMS, Patna.

Sample size and sampling technique

In the absence of documented evidence, we assumed that a 50% proportion of the study population would have an adequate readiness score. We required minimum sample size of 384 with 5% absolute precision and 95% CIs using openEpi software [10]. We used purposive sampling to achieve the sample size. We got a total of 411 responses.

Inclusion and exclusion criteria

We included all the faculty members, senior residents, junior residents, and nursing officers working at AIIMS Patna. Participants who were not directly involved in patient care and were denied to give consent for the study were excluded.

Study tool and technique

We used a pre-designed, pre-tested, and validated questionnaire by Reddy NK et al. [11] based on the "Operational Guidelines for Ayushman Bharat National Health Protection Mission (AB-NHPM)" [6] with minor modifications.

After taking written informed consent, the study tool was self-administered in English to the all-eligible HCWs. The faculty, residents, and nursing staff worked 24 hours a day, seven days a week in practically all main disciplines, serving a high number of poor patients and may be eligible for PMJAY.

The questionnaire had various sections. Section A comprised basic details of the HCWs, including designation and department of work. Section B comprised questions about knowledge of HCWs regarding PMJAY like initial payment to start the treatment, reimbursement of paid money, avail free medicines in OPD, and pre-authorization necessity to start treatment. Section C contained items related to the readiness of HCWs to implement PMJAY like initiating the treatment process, pre-authorization process, and documentation. The awareness part of the questionnaire had 10 items (on a three-point Likert scale 'yes,' 'no,' or 'do not know'). A 'yes' response received a score of 1, while a 'no' or 'do not know' response received a score of 0, and the sum of the scores was used to compute the participant's awareness score. The maximum score for the awareness section was 10. The readiness section had five items on a 5-component Likert scale, namely 'Not all,' 'occasionally on demand,' 'occasionally,' 'frequently,' and 'for all eligible cases.' The maximum attainable score for readiness was 25. The average content validity index (I-CVI) and average expert proportion of the tool items were 0.98 each, respectively [11].

The departments were broadly divided into surgical and medical specialties for ease of analysis. All wide and super specialties linked to surgery were included in the surgical department, while all other departments were included under the medical department.

Outcome variable

It is calculated using the participant's replies to the questions, testing their knowledge of PMJAY. The readiness score was determined by adding the responses to the items on the Likert scale that were relevant to readiness.

Statistical analysis

The collected information was entered and coded in MS Excel and analyzed using SPSS Version 22(SPSS Inc., Chicago, IL, USA). Results were tabulated and represented as figures wherever necessary. Descriptive analysis was conducted to describe the basic details of HCWs. The categorical variables like designation and department of HCWs were expressed as frequency and proportion, while continuous variables like awareness score and readiness score were expressed as mean (SD) after checking the normality. The difference in the awareness and readiness score across gender and departments of HCW was calculated by independent t-test, while the same across designation of HCW was assessed by one-way ANOVA followed by Tukey's post hoc analysis. Pearson's correlation 'r' was used to correlate awareness and readiness scores among HCW. Finally, simple linear regression was applied to assess the relationship between awareness and readiness scores. A p-value less than 0.05 was considered statistically significant.

Ethical consideration

This study has been approved by Institutional Ethics Committee, AIIMS, Patna (AIIMS/Pat/IEC/2021/784). We adhered to the principles of ethics throughout the study.

## Results

Out of 411 HCWs who participated, males (56.9%) outnumbered females (43.1%). Among the participants, about half of them were nursing officers (50.1%), followed by junior resident doctors (31.4%), senior resident doctors (13.6%), and faculty of medicine (4.9%). Almost 60.1% of respondents belonged to surgical specialties, and 38.9% were from medical specialties. Except for two participants, all others (409, 99.5%) had heard about the PMJAY scheme. However, only 6.1% of participants received some form of training on PMJAY. The overall mean (SD) awareness score was 5.52 (1.814), and the mean (SD) readiness score was 18.49 (4.498).

The mean (SD) awareness score for females (5.60 (1.811)) was slightly better than that of the males (5.46 (1.819)). However, the awareness score between males and females was not found to be statistically significant (p = 0.429). Similarly, the difference between mean (SD) awareness scores of medical (5.38 (1.801)) and surgical branches (5.62 (1.819)) was also not found statistically significant (p=0.187). The mean (SD) readiness score for males was 18.52 (4.89), and for females was 18.45 (3.922). No significant difference (p=0.086) was found between the mean (SD) readiness scores across medical (18.97 (4.55)) and surgical (18.19 (4.445)) departments (Table 1).

**Table 1:** Comparison of awareness and readiness scores across gender and department of HCW (n = 411). *P-value by independent sample t-test.

Variables	Category	N	%	Awareness scores		Readiness scores	
				Mean(SD)	P value*	Mean(SD)	P value*
Gender	Male	234	56.9	5.46 (1.81)	0.429	18.52 (4.89)	0.87
	Female	177	43.1	5.60 (1.81)		18.45 (3.92)	
Department	Medical	160	38.9	3.38 (1.80)	0.18	18.97 (4.55)	0.08
	Surgical	251	60.1	5.62 (1.81)		18.19 (4.44)	

Nursing officers (mean (SD) scores: 4.87 (2.1)) had significantly low awareness as compared to junior residents (mean (SD) scores: 6.07 (1.61)), senior residents (mean (SD) scores: 6.27 (1.72)), and faculty (mean (SD) scores: 6.6 (1.46)) (Table 2). When we applied the post hoc test to see the pairwise comparison of mean awareness score, the difference was significant between nursing officers and others (junior residents, senior residents, and faculty). No significant difference was observed among doctors (junior resident, senior resident, and faculty) (Table 3). When gender, different departments, and different HCW cadres were compared, no statistical significance in readiness score was identified among them.

**Table 2:** Comparison of awareness and readiness scores across designation of HCW (n = 411). * P-value by one-way ANOVA.

Designation	N	%	Awareness scores		Readiness scores	
			Mean(SD)	P value*	Mean(SD)	P value*
Nursing officer	206	50.1	4.87 (1.754)	0.001	18.13 (4.827)	0.057
Junior resident	129	31.4	6.07 (1.616)		18.47 (3.903)	
Senior resident	56	13.6	6.27 (1.721)		19.05 (4.704)	
Faculty	20	4.9	6.60 (1.465)		20.80 (3.328)	
Total	411	100	5.52 (1.814)		18.49 (4.498)	

**Table 3:** Pairwise comparison of mean awareness score across designation of study participants (post hoc test). *The mean difference is significant at the 0.05 level.

Pair	Mean difference in awareness score	95% CI of mean difference
Nursing Officer-Junior Resident	-1.19^*^	-1.69 to -0.71
Nursing Officer-Senior Resident	-1.39^*^	-2.05 to -0 .74
Nursing Officer-Faculty	-1.73^*^	-2.75 to -0.70
Junior Resident-Senior Resident	-0.19	-0.90 to 0.50
Junior Resident-Faculty	-0.53	-1.58 to 0.52
Senior Resident-Faculty	-0.33	-1.47 to 0.81

**Figure 1:**
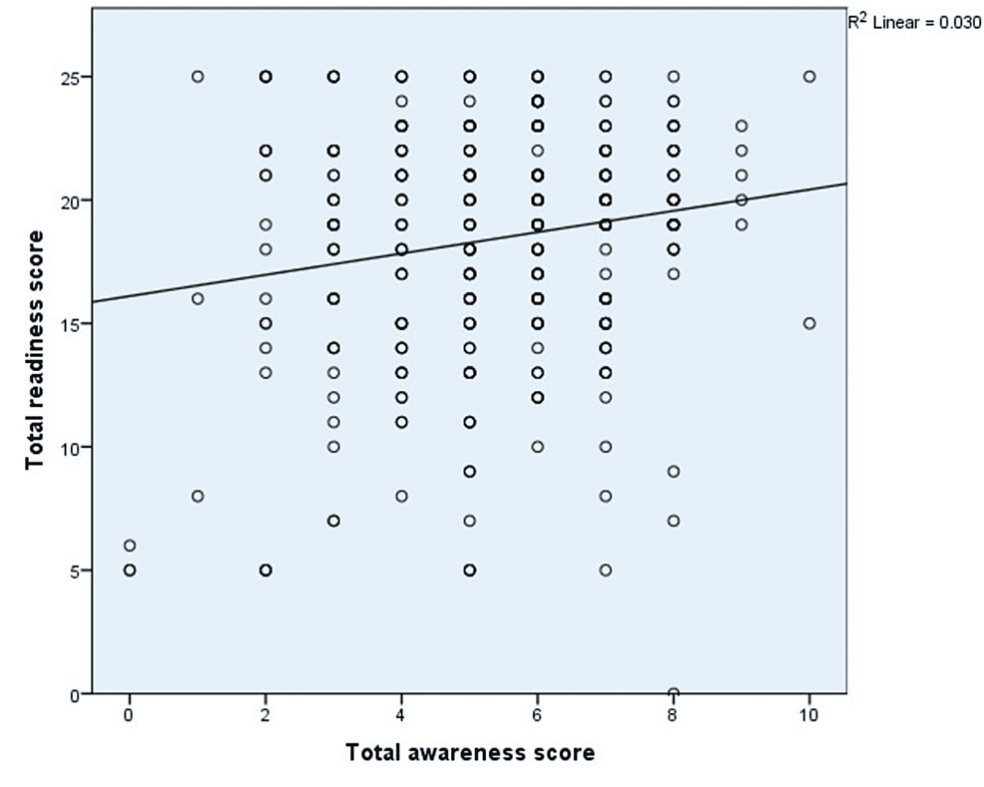
Relationship between readiness score and awareness score.

There was a statistically significant weak positive correlation between the awareness and readiness scores (r=0.174, p=0.001) (Figure 1).

To quantify the amount of change in preparedness score with change in awareness score, a simple linear regression analysis was used. Visual inspection of these two plots indicated a linear relationship between the variables. In addition, there was homoscedasticity and normality in the residuals. Therefore, the prediction equation was (total readiness score = 16.1+0.432*Total Awareness score), which shows that for every unit gain in awareness, there is an increase of 0.432 units in readiness, which is significant (p-value=0.001) (Table 4).

**Table 4:** Regression analysis summary for awareness score predicting readiness of the participants. Statistical results: R2= 0.03, R2 adjusted = 0.028, Durbin Watson value = 1.511, F (1, 409) = 12.78, p < 0.001.

Variable	B	Std. Error	95% CI	t	p
Constant	16.10	0.701	14.72-17.48	22.94	<0.001
Total Awareness Score	0.432	0.121	0.194-0.669	3.57	<0.001

## Discussion

Our cross-sectional study revealed that the overall mean (SD) awareness score was 5.52 (1.814), and the mean (SD) readiness score for implementing PMJAY was 18.49 (4.498). There was a significantly high awareness score among doctors compared to nursing officers. The relation between awareness score and readiness score was found to be statistically significant and showed a weak positive correlation (r=0.174, p=0.001). The linear regression model demonstrated a significant increase of 0.432 units in readiness for every unit increase in awareness score.

Our study's total awareness and readiness score was poor, which could be attributable to the fact that these HCWs did not receive any training. Nursing officers rated lower on awareness than faculty and residents, possibly due to their education level and a lack of programmatic information. There is a statistically significant rise in readiness scores with increasing awareness. This indicates how much better awareness can be achieved by doing training and awareness workshops before the program's launch. The mean awareness and readiness scores among medical and surgical branches were not statistically significant, according to a study conducted by Reddy NK et al. [11]. Faculty had a much higher awareness score than senior residents, in contrast to our study, which found no significant difference, even though faculty had a slightly higher mean awareness score than resident doctors. Similar to our study, they found that the relationship between awareness and readiness was correlated with Pearson's correlation of 0.206 and was statistically significant [11].

Earlier health insurance schemes, such as the Rashtriya Swasthya Bima Yojana (RSBY), also known as the National Health Insurance Programme, failed to take off, owing to a lack of commitment and involvement at the point of service delivery, which was once again overlooked [3]. As a result, it is vital to make sure HCWs are aware of the strategy and have been trained on it before it goes into effect, as this will help them be more prepared [12]. This can be further improved by including comments from HCWs to understand better the ground-level constraints that may develop during the scheme's implementation [13].

In India, the private healthcare sector has outpaced the public healthcare sector in terms of providing health services, and as a result, PMJAY has played an important role. However, according to data from India's similar healthcare systems, private healthcare practitioners may deviate from evidence-based practice and engage in unnecessary testing and treatment. In addition, when PMJAY gives financial assistance, unethical behavior may increase, placing the Government under more strain. Unethical behavior continues to be a concern at all levels, from service delivery to high-level program administrators and decision-makers [14,15].

## Conclusions

Even though there was enough information, education, and communication (IEC) about the PMJAY scheme's benefits, nursing officers' and doctors' average awareness score was relatively low. Resident doctors and nursing officers were less informed than faculty members. Nursing officers, residents, and faculty members all have the same level of readiness for implementing PMJAY in the eastern state of India. As the HCWs become more aware, there is an increase in readiness to implement PMJAY. The future scope of the study is to fill the knowledge gaps. Also, more training and awareness events should be held at all impaneled hospitals. Small sessions should be used to assess knowledge regularly. This may boost healthcare provider readiness, hence assisting in the scheme's planned aims being met. HCWs should be approached for their input on how to improve this knowledge so that we can meet our objectives.

Recommendations

To address the gaps in service delivery under PMJAY, public health administrators could arrange awareness activities in the form of continuing medical education (CME) or training sessions for HCWs. It is critical to ensure that all stakeholders are included. Stakeholders must evaluate and examine the program regularly. All impaneled hospitals should perform external audits of program execution to ensure that taxpayer money is not wasted.

Strengths and limitations of the study

Literature is scarce on this subject. This study examines HCW's awareness of PMJAY and their readiness to respond to it. This information will assist policymakers and health managers in taking the required actions to improve the scheme's implementation. This study is not without limitations. To begin with, our research was limited to a single center; therefore, the findings cannot be generalized. Second, the study participants may have been improperly distributed due to the adoption of a convenient sampling procedure.
